# Comparative transcriptome analysis and marker development of two closely related Primrose species (*Primula poissonii* and *Primula wilsonii*)

**DOI:** 10.1186/1471-2164-14-329

**Published:** 2013-05-14

**Authors:** Lu Zhang, Hai-Fei Yan, Wei Wu, Hui Yu, Xue-Jun Ge

**Affiliations:** 1 Key Laboratory of Plant Resource Conservation and Sustainable Utilization, South China Botanical Garden, Chinese Academy of Science, Guangzhou, 510650, China; 2 University of Chinese Academy of Sciences, Beijing, China

**Keywords:** Adaptive radiation, East Himalaya-Hengduan Mountains, EST-SSR, Single copy nuclear gene

## Abstract

**Background:**

*Primula* species are important early spring garden plants with a centre of diversity and speciation in the East Himalaya-Hengduan Mountains in Western China. Studies on population genetics, speciation and phylogeny of *Primula* have been impeded by a lack of genomic resources. In the present study, we sequenced the transcriptomes of two closely related primrose species, *Primula poissonii* and *Primula wilsonii,* using short reads on the Illumina Genome Analyzer platform.

**Results:**

We obtained 55,284 and 55,011 contigs with N50 values of 938 and 1,085 for *P. poissonii* and *P. wilsonii*, respectively, and 6,654 pairs of putative orthologs were identified between the two species. Estimations of non-synonymous/synonymous substitution rate ratios for these orthologs indicated that 877 of the pairs may be under positive selection (Ka/Ks > 0.5), and functional enrichment analysis revealed that significant proportions of the orthologs were in the categories DNA repair, stress resistance, which may provide some hints as to how the two closely related *Primula* species adapted differentially to extreme environments, such as habitats characterized by aridity, high altitude and high levels of ionizing radiation. It was possible for the first time to estimate the divergence time between the radiated species pair, *P. poissonii* and *P. wilsonii*; this was found to be approximately 0.90 ± 0.57 Mya, which falls between the Donau and Gunz glaciation in the Middle Pleistocene. Primers based on 54 pairs of orthologous SSR-containing sequences between the two *Primula* species were designed and verified. About half of these pairs successfully amplified for both species. Of the 959 single copy nuclear genes shared by four model plants (known as APVO genes), 111 single copy nuclear genes were verified as being present in both *Primula* species and exon-anchored and intron-spanned primers were designed for use.

**Conclusion:**

We characterized the transcriptomes for the two *Primula* species, and produced an unprecedented amount of genomic resources for these important garden plants. Evolutionary analysis of these two *Primula* species not only revealed a more precise divergence time, but also provided some novel insights into how differential adaptations occurred in extreme habitats. Furthermore, we developed two sets of genetic markers, single copy nuclear genes and nuclear microsatellites (EST-SSR). Both these sets of markers will facilitate studies on the genetic improvement, population genetics and phylogenetics of this rapidly adapting taxon.

## Background

Adaptive radiation, ‘the rise of a diversity of ecological roles and attendant adaptations in different species within a lineage’ is one of the most important processes bridging the gap between ecology and evolution [1]. Usually, the genetic divergence between species within adaptive radiations is very small, and only a handful of genes with large effects are responsible for differences in ecologically significant traits and reproductive isolation between species. Due to the lack of availability of molecular markers for rapidly evolving taxa, especially from nuclear genome, most plant molecular systematic studies on adaptive radiation have hitherto failed to provide resolved phylogenies. The same is true for speciation studies, which rely heavily on there being sufficient intraspecific genetic variation. Moreover, we still have little understanding of how divergent natural selection may have acted on the genomes of such species within the short evolutionary time span since their common ancestor [2].

Transcriptome analysis is not only an effective way to study gene expression in specific tissues at specific time, and it also provides unprecedented opportunities to address comparative genomic-level questions for non-model organisms. RNA-sequencing (RNA-seq) is an efficient new technology for large scale transcriptome investigations. With the rapid development of next-generation sequencing (NGS), RNA-sequencing becomes more efficient and less expensive, and is increasingly being used to study the evolutionary origins and ecology of non-model plants [3,4]. For instance, a large number of microsatellite markers or single-copy nuclear genes in yam (*Dioscorea alata*) [5], buckwheat (*Fagopyrum*) [6] and big sagebrush (*Artemisia tridentata*) [7] have been identified by making use of RNA-sequencing. Since RNA-sequencing is still somewhat expensive at present, few RNA-seq studies to date have included for more than one species at the same time [6,7]. However, comparative RNA-sequencing studies between closely related species can in principle not only provide additional genomic resources such as genus-specific SSR primers or single copy nuclear gene primers, but also give information about the processes of speciation or adaptive evolution, e.g. divergence time estimations, or detection of adaptive loci.

*Primula* with around 430 species, is one of the three great garden genera [8], and southwestern China, in which ca. 187 species of the genus are distributed, is its diversity centre [9,10]. In this region *Primula* shows a typical patterns of adaptive evolution and explosive speciation; however, research has been hampered by the fact that few *Primula* genomic resources are available. Up to now, only a few SSR primers from the three *Primula* species *P. vulgaris*, *P. obconica*, and *P. sieboldii* have been developed [11-13], and only one large EST collection, consisting of 5,651 ESTs generated from *Primula sieboldii* were available [13]. Paucity of genetic data such as genome sequences, transcriptome sequences and associated molecular markers has made *Primula* breeding or evolutionary analysis a challenging task.

*Primula* section *Proliferae* Pax, which contains ca. 25–30 species and is centred on southwestern China, is regarded as a taxonomically well-known group circumscribed by possession of numerous whorls of flowers [14]. Within this section, *Primula wilsonii* and *P. poissonii* are two closely related species with very similar morphological characters, and the two diagnostic characters used to distinguish them are the corolla structure and the aromatic fragrance of fresh leaves; for *P. wilsonii*, the fresh leaves are fragrant and corolla limbs are slightly opened, whereas, *P. poissonii* has no obvious fragrance and widely opened corolla limbs [14]. These closely related species represent a useful resource for addressing two questions: how did *Primula* species in southwestern China radiate within a short period of time, and what was the driving force underlying the process of rapid adaptive evolution? As the first step towards answering these questions, in this study, we obtained transcriptomes for *Primula poissonii* and *P. wilsonii* using the Illumina platform, and carried out a comprehensive analysis of them. Our aims were to 1) characterize the transcriptomes of *P. poissonii* and *P. wilsonii,* and increase the genetic resources available for *Primula* breeding or evolutionary analysis; 2) determine the evolutionary dynamics of the two species, including obtaining a divergence time estimation, signatures of adaptive evolution between the two species; and 3) discover genus-specific SSR markers and single-copy nuclear gene markers from both species.

## Results and discussion

### *De novo* assembly and functional annotation of contigs

**Figure 1:**
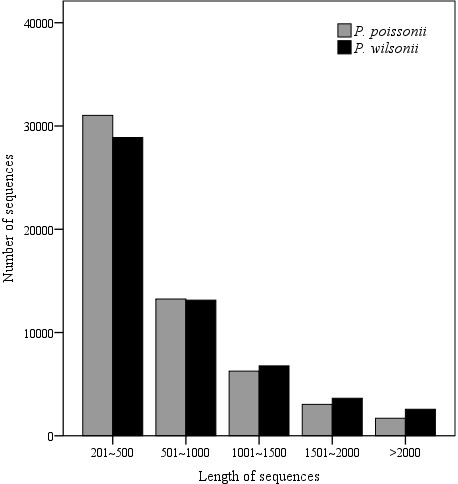
**Length distributions of contigs for two *Primula *species.** Grey bar, *P. poissonii*; black bar, *P. wilsonii*.

After cleaning of raw sequences, ca. 55 million 75-bp paired-end reads were obtained for both *P. poissonii* and *P. wilsonii*,. We obtained 55,284 contigs with a mean length of 655 and an N50 value of 938 for *P. poissonii*, and 55,011 contigs with a mean length of 722 and an N50 value of 1,085 for *P. wilsonii* (Table 1). Contig with lengths between 200 and 500 bp were overrepresented, making up about 56% of the total number of contigs for *P. poissonii,* and 53% for *P. wilsonii*, the next most abundant size class was 500–1000 bp, constituting about 24% and 24% of the total, respectively (Figure 1). To evaluate the quality of *de novo* assembly, we obtained a total of 16,346 peptide sequences from *Vitis.* For *P. poissonii*, 34,660 contigs were annotated to 13,800 (84.4%) *Vitis* proteins, of which, 6,869 (49.8%) proteins were covered for at least 70% of the full length. For *P. wilsonii*, 34,930 contigs were annotated to 13,955 (85.4%) *Vitis* proteins, of which, 7693 (55.1%) proteins were covered at least 70% of the full length. The GC content for *P. poissonii* and *P. wilsonii* sequences is 41.3% and 41.2%, respectively.

**Table 1:** **Summary of assembly and annotation results for *P. poissonii *and *P. wilsonii *using Trinity**

	***P. poissonii***	***P. wilsonii***
Total number of reads	55,056,996 × 2	55,468,564 × 2
Total number of contigs	55,284	55,011
Mean length of contigs	655	722
Median length of contigs	432	469
N50 value of contigs	938	1085
Length range of contigs	200 ~ 16,932	200 ~ 12,384
GC content	41.3%	41.2%
Contigs with BLASTX hit	36,239 (65.6%)	35,857 (65.1%)
Contigs with annotation	28,435 (51.4%)	28,302 (51.4%)

**Figure 2:**
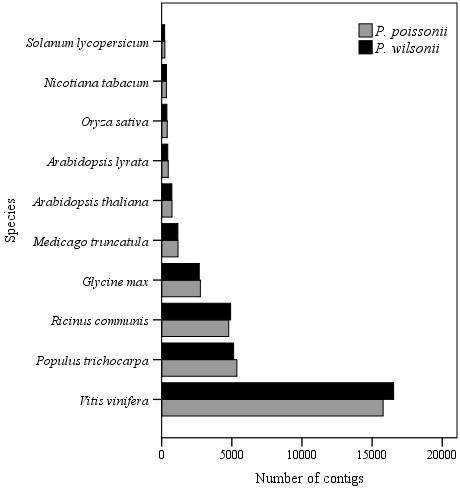
**Top-hit species distribution for sequences from two *Primula *species submitted BLASTX against the NCBI-nr. database.** Grey bar, *P. poissonii*; black bar, *P. wilsonii*.

**Figure 3:**
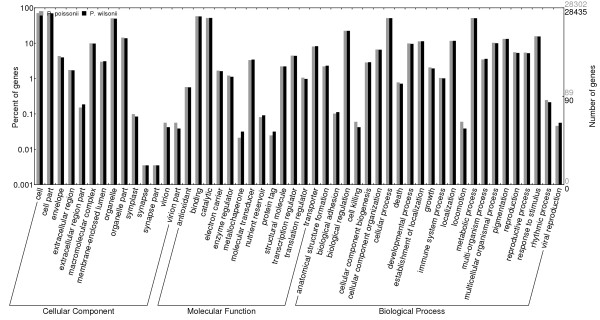
**Comparison of GO terms distributions between two *Primula *species.** Grey bar, *P. poissonii*; black bar, *P. wilsonii*.

In BLASTX homology research with the cutoff E-value set at 1E-6, 36,239 contigs (65.6%) for *P. poissonii* and 35,857 contigs (65.1%) *for P. wilsonii* gave hits. For both species, the three top-hit species were *Vitis vinfera*, *Populus trichocarpa* and *Ricinus communis* (Figure 2). A total of 28,435 (51.4%) and 28,302 (51.4%) contigs were assigned at least one GO terms for *P. poissonii* and *P. wilsonii*, respectively. For the biological process category, the two mostly highly represented terms among the 23 level-2 categories were cellular process and metabolic process; for the molecular function category, among the 13 level-2 categories, binding and catalytic activity were overrepresented; for the 14 level-2 categories in the cellular component category, cell, cell part and organelle were the most abundant terms (Figure 3). These categories were similarly distributed in both species.

### Orthologous contigs, substitution rates, and transcriptome divergence between two *Primula* species

We identified 28,482 pairs of putative orthologous contigs between *P. poissonii* and *P. wilsonii* using the reciprocal best hit method with BLASTN algorithm. After incorporating the *Vitis* peptide sequences [15], 7,006 pairs of putative orthologs were obtained using the RBM triangulation method [16]. This reduction in ortholog numbers was caused mainly by the exclusion of the relatively young orthologs specific to *Primula,* which were discarded as being low similarity to *Vitis*. After excluding alignments with unexpected stop codons, lengths less than 150 bp or Ks values above 0.1, 6,654 pairs of orthologs were retained for subsequent analysis.

Using the *Vitis* proteins as reference, the coding regions of 6,654 pairs of orthologs from *P. poissonii* and *P. wilsonii* were extracted, in some cases, 5′-UTR (1,315 pairs of orthologs) or 3′-UTR (2,051 pairs of orthologs) were also determined. The average genetic divergence of coding regions between the two *Primula* species is 0.011 ± 0.007 according to the K2P model. The genetic divergence between the two species is 0.019 ± 0.017 for 5′-UTR and 0.018 ± 0.013 for 3′-UTR regions. The accelerated substitution rate observed in the 5′UTR and 3′ UTR relative to the coding region, is indicative of relaxed functional constraint on the evolution of the UTR than on the coding region at the genome level, which is consistent with the evidence from other model species-pairs [17].

Among the 6,654 pairs of orthologs between *P. poissonii* and *P. wilsonii*, 165 pairs were identical, 1,327 pairs had only either synonymous or nonsynonymous substitutions, and 5,162 pairs had both types of substitutions, for which the Ka/Ks ratio were calculated. The mean values of Ka, Ks, and the Ka/Ks ratio of all orthologous pairs were 0.007 ± 0.005, 0.027 ± 0.017 and 0.322 ± 0.324, respectively. Of the 5,162 pairs of orthologs, 233 pairs with a Ka/Ks value > 1 were found. Taking a more appropriate threshold of 0.5 for the Ka/Ks ratio as an indicator of positive selection [18], 644 pairs with a Ka/Ks value between 0.5 and 1 were also found.

**Figure 4:**
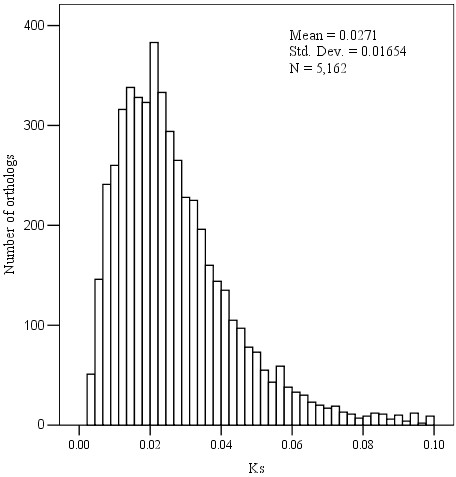
**The Ks distribution of orthologs between *P. poissonii *and *P. wilsonii*, their divergence is shown by the peak of Ks at 0.03 ± 0.017.**

Peaks in the Ks value distribution of orthologs between closely related species often indicates speciation events [19], and this approach has been successfully used in the inference of such events [20]. In this study, a peak of Ks distribution between *P. poissonii* and *P. wilsonii* was observed at 0.027 ± 0.017 (Figure 4). The low level of Ks between *P. poissonii* and *P. wilsonii* indicated their close relationship and confirmed the previous taxonomic treatment. Based on the data derived from ESTs of Asteraceae and several model plants provided by Kane [21] (personal communication), we found a mean Ks value of 0.03 ~ 0.10 between congeneric species. According to these criteria, the differentiation between *P. poissonii* and *P. wilsonii* is obviously very recent.

The peak synonymous rates (Ks) for orthologous transcript pairs can be used to estimate the times of divergence between species. To obtain a rough estimate of the divergence time (T) between *P. poissonii* and *P. wilsonii*, we followed the simple formula: T = K/2r [22], where r is the mean rate of synonymous substitution, and is considered to be 1.5E-8 substitutions/synonymous site/year for all dicots [23]; K is genetic divergence expressed in terms of mean number of synonymous substitutions between orthologs. The age of the speciation event between *P. poissonii* and *P. wilsonii* is approximately 0.90 ± 0.57 Mya, which falls between the Donau and Gunz glaciation in the Middle Pleistocene. Bearing in mind disputes about the substitution rate [24], this divergence time is only an appropriate estimate based on the coding region of orthologous genes, nonetheless, it is useful because there is as yet no adequate fossil dating the divergence of the two *Primula* species.

### Functions under positive selection and implications for adaptive evolution between two *Primula* species

In enrichment analyses, we categorized the orthologs into two datasets: a test dataset with Ka/Ks > 0.5, and a reference dataset with Ka/Ks < 0.5. In an analysis of GO terms with at least five hits, 20 GO-terms annotated to 98 pairs of orthologs were found to be over-represented (Fisher’s exact test, *P*-value < 0.05) in the test dataset (Table 2, Additional file 1: Table S1). For the 98 selected genes, we used BLASTX search to find their orthologous genes in Arabidopsis, and the results showed that the genes with function in DNA repair, stress resistance were overrepresented (Tables 2 and 3). Among the candidate genes under positive selection with Ka/Ks > 0.5, almost one-quarter of them were involved in the DNA repair. DNA repair is essential for maintenance of genomic stability in all organisms. For instance, in our study, the ortholog pw11166, and pw24180 were found to be homologous to *Ku70* and *Ku80*, which are involved in the repair of DNA double-strand breaks (DBSs) by non-homologous end joining (NHEJ) [25]; other orthologs, pw42431, pw06163, pw54400 were homologous to *SMC5*, *ETG1*, *ROR1*, respectively, which are all involved in DNA repair by homologous recombination [26-28]. The finding that gene families *Ku*, and *SMC* have been under positive selection gives an indication of why *P. poissonii* adapted to the habitats of higher altitude and ionizing radiation than did *P. wilsonii*.

**Table 2:** Gene Ontology terms significantly over-represented in the test dataset versus the reference set

**GO ID**	**GO term**	**P-value (Fisher’s exact test)**	**Frequency in test set**	**Frequency in reference set**
	**Biological Process**			
GO:0007059	chromosome segregation	0.05	3	3
GO:0016790	thiolester hydrolase activity	0.03	6	11
GO:0035966	response to topologically incorrect protein	0.03	3	2
GO:0006281	DNA repair	0.04	14	43
GO:0006289	nucleotide-excision repair	0.01	4	2
GO:0032012	regulation of ARF protein signal transduction	0.05	3	3
GO:0006544	glycine metabolic process	0.05	3	3
GO:0042542	response to hydrogen peroxide	0.04	5	8
GO:0022613	ribonucleoprotein complex biogenesis	0.03	11	28
GO:0071843	cellular component biogenesis at cellular level	0.03	14	41
GO:0006979	response to oxidative stress	0.04	15	47
GO:0006974	response to DNA damage stimulus	0.02	16	47
GO:0044085	cellular component biogenesis	0.05	27	102
	**Molecular Function**			
GO:0008173	RNA methyltransferase activity	0.05	5	9
GO:0004221	ubiquitin thiolesterase activity	0.03	4	5
GO:0003960	NADPH: quinone reductase activity	0.03	3	2
GO:0016790	thiolester hydrolase activity	0.03	6	11
GO:0035091	phosphatidylinositol binding	0.05	5	9
	**Cellular Component**			
GO:0015934	large ribosomal subunit	0.03	5	9
GO:0005802	trans-Golgi network	0.04	3	2

**Table 3:** **Partial list of candidate orthologs under positive selection between *P. wilsonii *and *P. poissonii***

***P. wilsonii***	***P. poissonii***	**Ka/Ks**	**Arabidopsis thaliana gene accession**	**Descriptions**
pw11166	pp53622	1.902	AT1G16970	*KU70*, atp-dependent dna helicase 2 subunit ku70
pw24180	pp53932	1.201	AT1G48050	*KU80*, atp-dependent dna helicase 2 subunit ku80-like
pw42431	pp10104	2.016	AT5G15920	*SMC5*, structural maintenance of chromosomes protein
pw06163	pp48369	1.087	AT2G40550	*ETG1*, mini-chromosome maintenance complex-binding
pw54400	pp05772	1.131	AT2G24490	*ROR1*, replicon protein a2
pw23141	pp39494	1.073	AT4G31870	*GPX7*, glutathione peroxidase
pw12283	pp40105	1.726	AT1G13180	*ARP3*, actin-related protein 3
pw37795	pp02860	2.329	AT1G11755	*LEW1*, nogo-b receptor-like
pw10616	pp13461	3.711	AT1G45976	*SBP1*, s-ribonuclease binding protein 1
pw09970	pp42238	1.106	AT3G11050	*FER2*, ferritin subunit precursor
pw13953	pp20861	0.777	AT3G54340	*AP3*, mads-domain transcription factor
pw42171	pp17454	1.099	AT2G47460	*MYB12*, transcription factor myb12

Some orthologs related to abiotic stress were also found to be positively selected. For example, pw23141 is homologous to *GPX7*, which regulates cellular photooxidative tolerance and immune response [29]; pw12283 is homologus to *ARP3,* related to light-induced stomatal opening [30]; pw37795 is homologous to *LEW1*, the product of which catalyzes the biosynthesis of dolichol [31] and confers acclimation to drought stress, which may partially explain why the two *Primula* species were able to inhabit habitats with different level of aridity; pw10616 is homologous to *SBP1* and pw09970 is homologous to *FER2*, which are involved in the cadmium stress [32] and iron deficiency [33], respectively; these results shed further light on how the two *Primula* species differentially adapted to extreme environments. In addition, two positively selected genes are worth notice, one gene pw13953, is homologous to *AP3*, a key component in the ABC mode of flower development [34], and may provide a clue about the origin of the differences in corolla structure between *P. poissonii* and *P. wilsonii*; the other gene pw42171, is homologous to *MYB12,* which functions as a *R2R3-MYB* transcription factor in phenylpropanoid biosynthesis [35], also may give some hints on the leaf fragrance differentiation between *P. poissonii* and *P. wilsonii*.

Overall, in this study, we detected a dozens of gene under positive selection between the *Primula* species pairs, and these findings will not only shed light on how differentiations between two *Primula* species occurs, but also open the door to increased understanding of how plants living in plateau environments adapt to different characteristics of high altitude, such as strong radiation, aridity and so on.

### Identification of microsatellites and single copy genes

**Figure 5:**
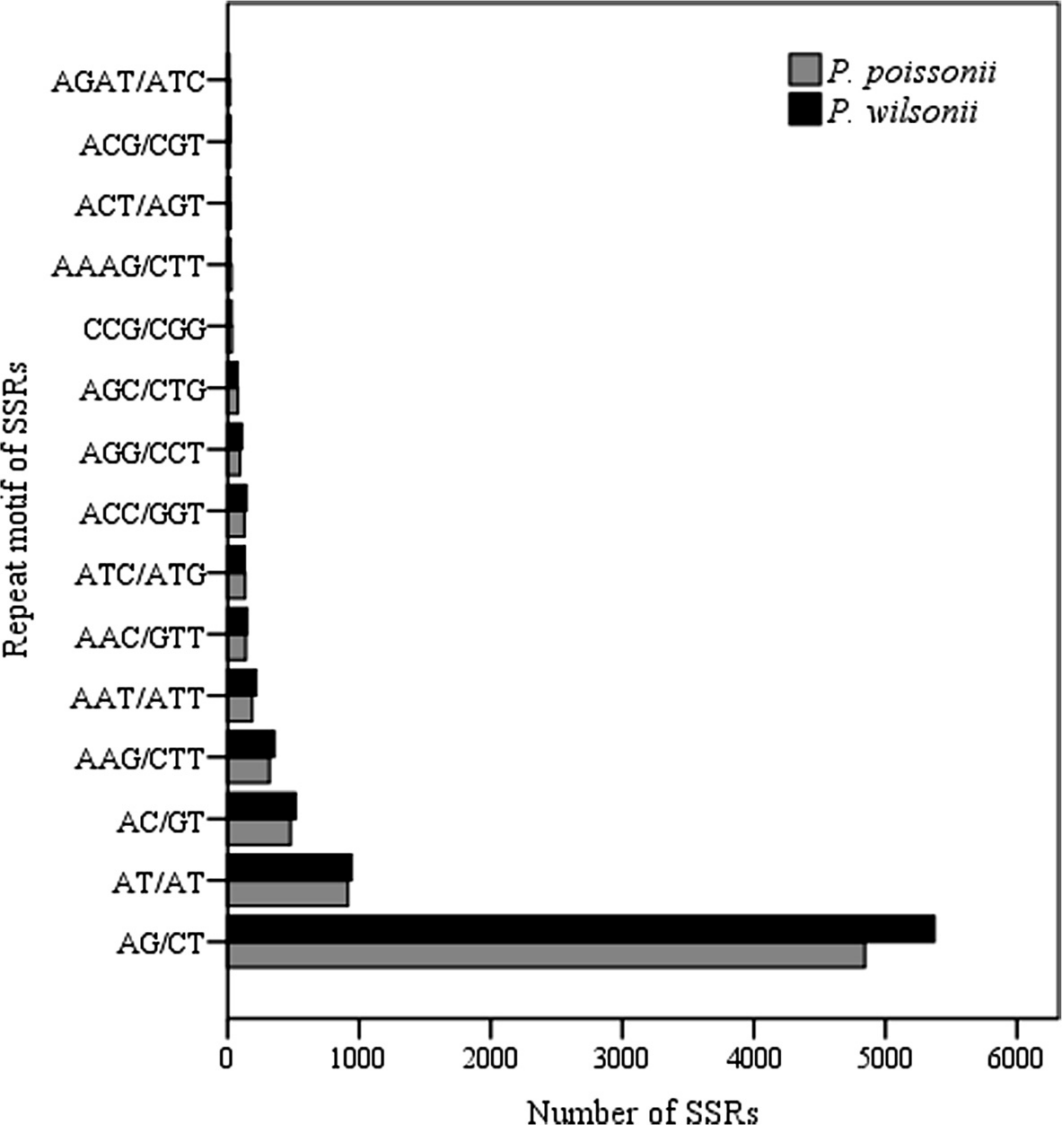
**Comparison of the distribution of SSR repeat motifs between two *Primula *species.** Grey bar, *P. poissonii*; black bar, *P. wilsonii*.

**Figure 6:**
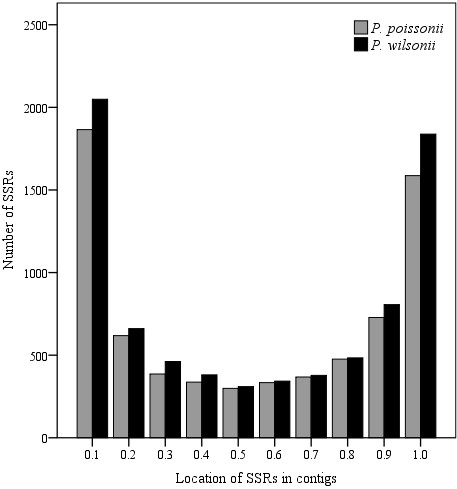
**The location distribution of SSRs for the two *Primula *species; the location is defined by the ratio of the start site position to the total length of the SSR-containing contigs.** Grey bar, *P. poissonii*; black bar, *P. wilsonii*.

**Figure 7:**
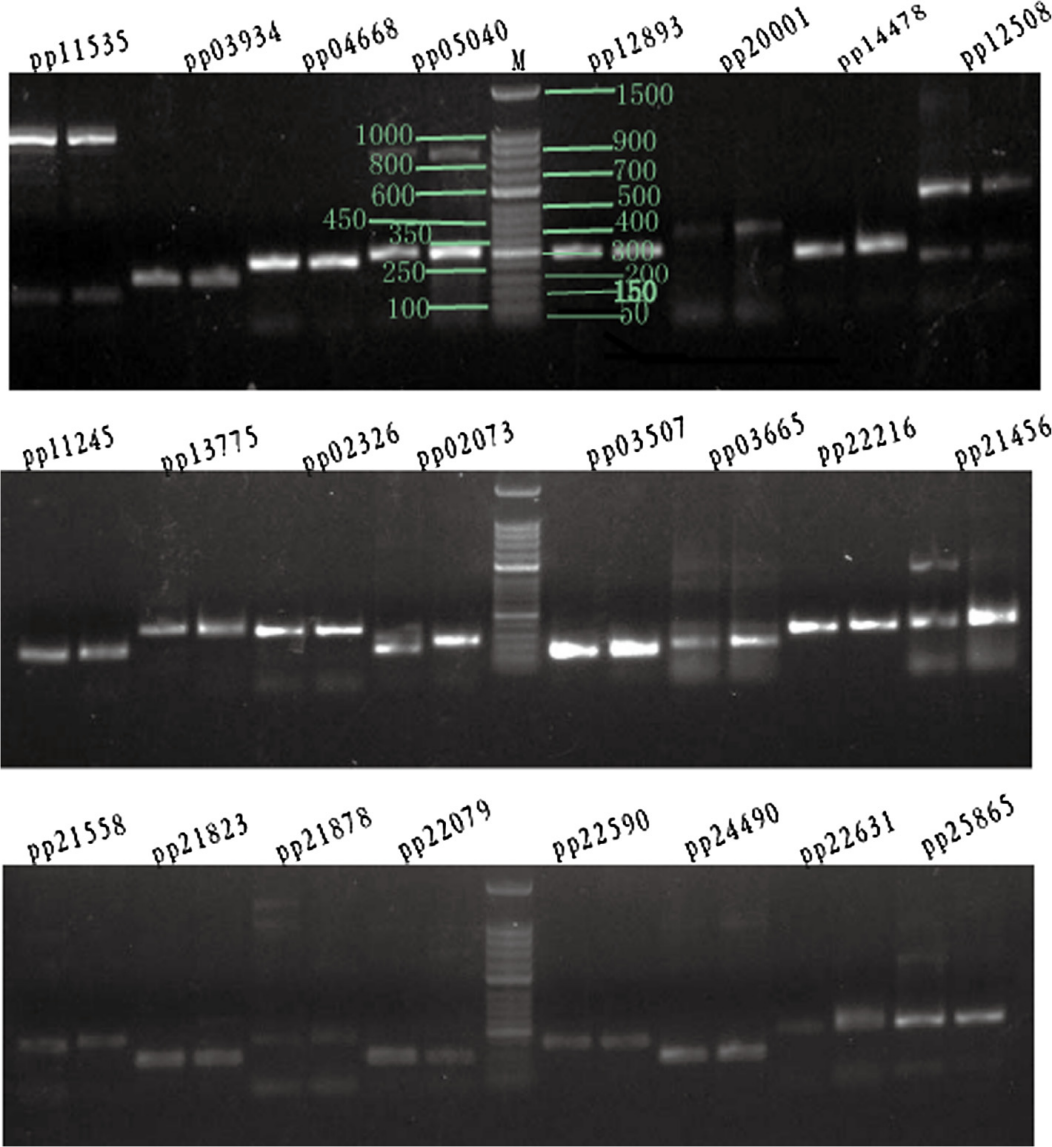
**Validation of a subset of the microsatellite primer pairs for the two *Primula *species by agarose-gel profiling.** Results are shown for one individual from each species with pairs name on top; the band profiles for the 50 bp maker ladder are illustrated; additional bands over 300 bp for some primer pairs are non-specific products.

Usually, SSR markers derived from expressed sequence tags (EST-SSRs) are more transferable between species than random genomic SSRs, and they are more advantageous for revealing adaptive differentiations at the population level. Traditional strategies for SSR marker development are labour-intensive and costly. In the case of *Primula*, up to now, only a few microsatellite primers have been available for *Primula obconica*, *P. sieboldii* and *P. vulgaris*[11-13], and this has impeded genetic analysis of this important garden plant. Based on the two *Primula* transcriptomes, 7,571 and 8,272 SSRs were found in *P. poissonii* and *P. wilsonii*, respectively. The most abundant repeat types were dinucleotides followed by trinucleotides (Table 4). The dominant classes of sequence repeat in the contigs were AG/CT, AT/TA and AC/GT, followed by AAG/CTT repeats (Figure 5), and most SSRs located close to the ends of contigs and were not suitable for primer design (Figure 6). In order to maximize the universal applicability of markers developed in this study and hence reduce their cost, we searched for SSRs in the 6,654 pairs of putative orthologous contigs, and found 1,207 SSRs distributed among 1,073 pairs of contigs (Table 4). Taking only those with a repeat-unit length of at least 16 bp, 421 pairs of SSRs contained in 342 pairs of orthologs were selected for primer design, and 54 pairs of sequences with conserved, sufficiently long flanking sites were used to design primers successfully (Additional file 1: Table S2). To evaluate the reliability of these primers, we tested 36 out of the 54 pairs and 24 pairs produced clear fragments with the expected sizes in both *Primula* species (Figure 7).

**Table 4:** **Summary of microsatellite loci in *Primula poissonii *and *P. wilsonii***

**Dataset**	**Contigs containing SSRs**	**SSRs**	**Di-nucleotide repeats**	**Tri-nucleotide repeats**	**Tetra-nucleotide repeats**	**Penta-nucleotide repeats**	**Hexa-nucleotide repeats**
*P. poissonii*	6363	7571	6245	1158	88	51	29
*P. wilsonii*	6981	8272	6833	1249	85	65	40
ortholog pairs	1073	1207	1046	157	3	1	0
SSRs (repetitive units >16 bp)	342	421	288	89	3	1	0

One noteworthy fact about the SSR primer development based on the Illumina platform is the lower proportion of contigs suitable for primer design compared with Sanger sequencing, in our study, 367 out of the 421 pairs of contigs with SSRs were excluded from primer design because they had insufficient flanking regions caused by NGS assembly algorithms or sequencing [36]. As an alternative, the 454/Roche sequencing platform, which delivers longer reads, has promise as a way of reducing bias.

Using the APVO gene sets [37] to carry out TBLASTN queries against our *Primula* dataset orthologs, 612 of the APVO genes were found to give hits against orthologous contigs between *P. poissonii* and *P. wilsonii* over at least 600 bp; these are most likely to be single copy genes in the two *Primula* species. When we set a threshold identity of 75% with *Arabidopsis thaliana* and specified facultative intron sizes not less than 300 bp in *Arabidopsis thaliana*, we were successful in obtaining primers for 111 of the 612 APVO genes (Additional file 1: Table S3); we randomly selected four primer pairs to test, and of these, three of the pairs amplified successfully and when the products were sequenced directly, they yielded the expected gene model, and only one primer pair failed due to the presence of extremely long intron variants, which made the products unsuitable for Sanger sequencing.. The availability from this study of dozens of single copy nuclear genes with heterogeneous rates of variation, will undoubtedly facilitate phylogeny resolution for the radiative *Primula* species*,* and open a doorway to understanding the dynamics of speciation using a population genetics approach.

We developed sets of two types of molecular markers, and these two widely-used marker types, each of which has its own advantages, were applied for characterizing population structure, parentage analysis, genotyping, gene flow inferences and phylogenetic construction. The large number of novel single nuclear gene will greatly increase the resolution of phylogenetic reconstruction for this adaptive taxon. Moreover, these markers with their diverse evolutionary rates will provide unprecedented opportunities to answer the following important questions: What demographic histories underlie the phylogeographic patterns of *Primula* species? Which evolutionary forces drive the explosive radiation of *Primula* species in the extreme habitats?

## Conclusions

In this study, we characterized the transcriptomes for the two *Primula* species, and obtained an unprecedented amount of genomic resources for these important garden plants. Evolutionary analysis of these two species not only yielded a more precise divergence time, but also provided some novel insights into how differential adaptations occurred in extreme habitats. In addition, we developed two sets of genetic markers of popular types, single copy nuclear genes and nuclear microsatellites (EST-SSR). These marker sets will facilitate studies on the genetic improvement, population genetics and phylogenetics of this rapidly adapting taxon.

## Methods

### Plant material, RNA extraction and sequencing

*Primula poissonii* (2n = 22) is widespread in the mountain areas of northwest Yunnan and southwest Sichuan, China. It inhabits alpine meadows at an altitude of 3200–3500 m. *Primula wilsonii* (2n = 22) is distributed in central Yunnan and grows on open lands in evergreen broad-leaved forest at an altitude of ca. 2500 m [8]. During July 2011, we sampled *P. poissonii* from Zhongdian plateau in northwest Yunnan (28°06'55.24"N, 99°47'48.49''E, alt. 3314 m), and *P. wilsonii* from Ailao Mountain in central Yunnan (24°32'34.47''N, 101°01'41.48''E, alt. 2450 m), respectively, and fresh leaves and whole flowers of multiple individual plants for both species were stored in RNAlater solution (Takara Biotechnology Co. Ltd., Dalian, China) to preserve the RNA state for use immediately after harvesting. After mixing an approximately equivalent weight of fresh leaves and flowers, total RNA was extracted using a modified CTAB method and precipitated with 5 M LiCl_2_ at -20°C overnight, and the resulting RNA pellets were suspended in about 100 μl DEPC-treated water. After assessing RNA quality by means of electrophoresis and an Eppendorf AG 2231 BioPhotometer Plus (Hamburg, Germany), quantified total RNA (concentration ≥ 100 ng/μL; rRNA ratio ≥ 1.5) were delivered to The Beijing Genome Institute (Shenzhen, China) for further treatments. The cDNA library for transcriptome sequencing was prepared using a cDNA Synthesis Kit (Illumina Inc., San Diego, CA, USA) following the manufacturer’s recommendations. The cDNA library was then sequenced using a HiSeq2000 (Illumina Inc, San Diego, CA, USA) to obtain short sequences of 90 bp from both ends of each cDNA.

### Sequence cleaning, assembly, contig annotation

Raw reads were firstly subjected to cleaning by removal of adaptors, reads with too many Ns, and reads with quality scores lower than 20. The cleaned reads were assembled *de novo* using Trinity [36] with the default parameters and contigs with length less than 200 bp were discarded due to a low annotation rate [38]. The filtered reads for *P. poissonii* and *P. wilsonii* were deposited in the NCBI Sequence Read Archive (SRA) under the accession number SRR629689 and SRR640158, respectively.

Functional annotation was implemented using the online program Blast2GO v.2.6.0 [39]. All the assembled contigs were firstly subjected to BLASTX against the NCBI’s non-redundant protein database with an E-value threshold of 1E-6. The predicted gene name for each contig was assigned according to the best BLASTX hit. Gene Ontology [40] terms were retrieved from BLASTX hits at E-value threshold 1E-6. Finally, the distributions of level-2 GO terms for all contigs were plotted with the program WEGO [41]. In addition, we download the *Vitis vinfera* proteome [15], and queried against all the assembled contigs using TBLASTN with an E-value threshold of 1E-10 [42].

### Identification of orthologous contigs and estimation of substitution rates

The reciprocal best matches (RBM) method [43] is widely used for identifying orthologs, and a modified version program RBM triangulation [16], allows a third species to be incorporated, which can increase reliability and detect large numbers of conserved orthologs, so we used the following approach for this study. First, we used BLASTN with the RBM method to find orthologs between the two *Primula* species setting the E-value cutoff at 1E-10. Next, to avoid misspecification caused by the absence of a true ortholog from either *Primula* species, the third species *Vitis* was added as positive control. All the reciprocal best hit orthologs were subjected to BLASTX against the *Vistis* peptide sequences at a threshold E-value of 1E-10, and only those pairs of orthologs with the same reciprocal best hit with *Vitis* were kept.

With the *Vitis* peptide sequence as reference, 5′UTR or 3′UTR sequences for some *Primula* contigs were determined. According to the best-match *Vitis* peptide sequences, the coding region sequences (CDS) of all the contigs were extracted with custom Perl scripts, and subsequently aligned using the MUSCLE algorithm [44] implemented in MEGA5 [45]. Alignments with unexpected stop codons, or less than 150 base pairs in length, were discarded after checking manually. For the remaining orthologs, synonymous substitution rates (Ks) and non-synonymous rates (Ka) were estimated using a maximum-likelihood method [46] implemented by yn00 in the PAML toolkit [47]. For the closely related species pair, *P. poissonii* and *P. wilsonii*, orthologs with Ks > 0.1 were excluded to avoid paralogs [48]. Divergence in the CDS sites and UTRs were calculated using the K2P model with a custom Perl script.

On the basis of the Ka/Ks value, setting a threshold at 0.5, the orthologs were sub-categorized into two dataset: a test set with Ka/Ks above 0.5, and a reference dataset with Ka/Ks value less than 0.5. The significance of the difference in GO term abundance between the two datasets was tested using the Fisher’s exact test with the GOSSIP package [49] implemented in BLAST2GO V.2.6.0 [39].

### Simple sequence repeats (SSRs) identification and mining of single copy nuclear genes

The program MISA (http://pgrc.ipk-gatersleben.de/misa/) [50] was used to identify and localize microsatellite motifs in the two *Primula* species, and only those contigs with motifs containing at least five repeats were selected. The alignments of 6,631 pairs of orthologs were extracted as the input file for the MISA program. Using the detailed information on SSR loci obtained from the output of the MISA program, primers for each SSR-containing sequence with a repetitive at least 16 bp in length were designed with Program Primer Premier 5 (PREMIER Biosoft Int., Palo Alto, CA). To validate the SSRs identified *in silico* identified SSRs, primer pairs shared between the two *Primula* species were synthesized (Invitrogen Trading Shanghai Co., Ltd, Shanghai, China), and amplified with one individual of each species as templates. PCRs were performed in a 25 μl volume containing 25 ng of template genomic DNA. The PCR reactions were carried out under the following conditions: initial denaturation at 95°C for 2 min, 35 cycles at an annealing temperature ranging from 45 ~ 60°C for 50 s, and a final extension at 72°C for 10 min. The PCR products were checked on 1.5% agarose gel.

Duarte et al. (2010) identified about 959 sets of single copy nuclear genes shared by *Arabidopsis*, *Populus*, *Vitis* and *Oryza* (known as APVO genes). We extracted the protein sequences encoded by the APVO gene from the TAIR10 database and queried them against the *Primula* orthologous EST database using TBLASTN [42] with a threshold E-value of 1E-10. All the queries with hits were considered to be single copy nuclear genes in the *Primula* species, and the consensus contigs of best- matched orthologous pairs of the two *Primula* species were extracted for degenerate primer design using the SeqMan 5.0 program (DNASTAR Inc, Madison, WI, USA). The consensus sequences were queried against the *Arabidopsis thaliana* protein database using BLASTX with an identity threshold of above 0.75, then subjected to exon-anchoring and intron-spanning primer design according to the corresponding Arabidopsis thaliana gene models with Program Primer Premier 5 (PREMIER Biosoft Int., Palo Alto, CA, USA). To validate these primers, four of them were randomly chosen to amplify with genomic samples from one individual of each of the two *Primula* species, and the products were sequenced*.*

## Competing interests

The authors declared that they have no competing interest.

## Authors’ contributions

XJG and WW conceived and designed the project. WW and HFY performed the experiments. LZ, WW, XJG analysed and interpreted the data. LZ, WW and XJG drafted the manuscript. HFY and HY revised the manuscript. All authors read and approved the final manuscript.

## Supplementary Material

Additional file 1 **Table S1.** Candidate orthologs under positive selection between *P. wilsonii* and *P. poissonii*. **Table S2.** Characteristics of conserved microsatellites primers based on two Primula orthologs. **Table S3.** Characteristics of primers for Primula-specific single copy nuclear genes homologous to APVO gene sets. Click here for file
